# High depressive symptomatology reduces emotional reactions to pictures of social interaction

**DOI:** 10.1038/s41598-024-51813-1

**Published:** 2024-01-13

**Authors:** Kíssyla Christine Duarte Lacerda, Fabiana Cristina de Oliveira Souza, Cassia Regina Vieira Araújo, Bruna Eugênia Ferreira Mota, Pedro Maria Guerra Muñoz, Willian Berger, Liliane Vilete, Eduardo Bearzoti, Gabriela Guerra Leal Souza

**Affiliations:** 1 Laboratory of Psychophysiology, Department of Biological Sciences, Federal University of Ouro Preto, Ouro Preto, 35400000 Brazil; 2 School of Nutrition, Federal University of Ouro Preto, Ouro Preto, 35400000 Brazil; 3 Department of Clinical Psychology, Universidad de Granada, 18010 Granada, Spain; 4 Institute of Psychiatry, Federal University of Rio de Janeiro, Rio de Janeiro, 22290-140 Brazil; 5 Department of Statistics, Federal University of Ouro Preto, Ouro Preto, 35400000 Brazil

**Keywords:** Neuroscience, Physiology, Psychology

## Abstract

Individuals with severe depressive symptoms present diminished facial expressions compared to healthy individuals. This reduced facial expression, which occurs in most depressive patients could impair social relationships. The current study sought to investigate whether pictures with social interaction cues could elicit different modulations of facial expressions and mood states in individuals with depressive symptoms compared to healthy individuals. A total of 85 individuals were divided into depressive and non-depressive groups based on their beck depression inventory scores. Participants viewed pictures containing neutral (objects), affiliative (people interacting socially), and control (people not interacting) scenes. Electromyographic signals were collected during the entire period of visualization of the blocks, and emotional questionnaires were evaluated after each block to assess sociability and altruism (prosocial states). In non-depressed individuals, affiliative pictures increased the activity of the zygomatic muscle compared to both neutral and control pictures and reduced fear of rejection compared to neutral pictures. During the visualization of the affiliative block, zygomatic major muscle activation was higher and fear of rejection was lower in the non-depressive individuals than in the depressive. These effects reflected the low expressions of smiling and sociability to affiliative pictures in depressive individuals. These findings highlight the importance of smiling and prosocial states in social interactions, especially in these individuals.

## Introduction

One of the most important evolutionary characteristics of human beings is their social nature. The ability to develop social bonds in the environment in which we live largely depends on the ability of individuals of the same species to recognize and accurately interpret the emotional state of other group members^1,2^. The ability to correctly identify emotions, as well as to demonstrate emotions through facial expressions, is crucial for communication^3,4^. Facial expressions are, therefore, a major contributor to social connections, as they not only express emotional states but also convey intentions^5,6^. This was demonstrated by Bradley et al.^7^, in which pictures of families and babies were rated as highly pleasant, and were associated with the activation of neural systems that lead to closeness and evoked greater activity of the zygomatic major muscle (related to a smile) compared to the activities evoked by neutral or unpleasant pictures. Likewise, social bonding stimuli, such as pictures of people interacting, are perceived as more pleasant (high valence) and more arousing (greater activation) than pictures depicting the same people not making direct social contact^8^. Pictures with social interaction cues were recently shown to promote larger EMG activation of the zygomatic major muscle in healthy individuals compared to that for pictures without social interaction. Pictures with social interaction cues also increased the expectation of closeness and reduced fear of rejection in these individuals^9^.

People with depression have difficulties in social interaction processes^10–13^ and have differentiated muscle activity in response to emotional stimuli. Evidence indicates that individuals with depressive symptoms express fewer facial expressions than those without these symptoms, except when the expression is related to sadness^13,14^. Low facial responsiveness occurs mainly in the activity of the zygomatic major muscle associated with happy facial expressions^4,15–18^. Low facial expressiveness, together with deficits in the recognition of facial emotions, can compromise interaction initiation and subsequent development^5,6^. Consequently, individuals with depression have fewer interactions and greater social rejection by non-depressed individuals. Thus, these individuals may assess themselves and be judged by others to be less socially competent^13^.

Although individuals with depression show reduced zygomatic activity when viewing pleasant pictures in general^4,15–18^, studies have not yet investigated the differences in the EMG reactivity of the zygomatic major in these individuals with pleasant pictures in a context of social interaction. Therefore, we investigated whether the smiling facial expression of individuals with high levels of depressive symptoms differed from those of individuals without depressive symptoms when viewing pictures with and without social interaction clues, with the same valence and activation, when the only difference between pictures is the presence or absence of social interaction cues. We also evaluated whether, as observed in individuals without depression, social interaction cues were relevant enough to increase the emotional states of sociability and altruism in individuals with depression.

We believe that this is the first study to compare the reactivity of the zygomatic major muscle, affiliative state and altruism behavior evoked by visual stimuli that portray the same people, in the same background scene and environment, with valence and activation pairing, where the only difference between the stimuli is the context of social bond (pictures with and without social interaction) in individuals with and without depressive symptoms. Therefore, we aimed to investigate whether social interaction pictures differently modulated the facial EMG reactivity of the major zygomatic muscle, affiliative state, and altruistic behavior between individuals with and without depression. We hypothesized that the group with depression would exhibit less zygomatic muscle reactivity and lower levels of closeness expectation and altruism when viewing affiliative pictures compared to those in the group without depression. We also hypothesized that the group with depression would not show altered fear of rejection, while the group without depression would show a lower level when viewing interaction pictures. Our general hypothesis was that individuals with depression are less sensitive to the social interaction cues displayed in pictures of social interaction stimuli (affiliative pictures) compared to non-social interaction (control pictures) and neutral image stimuli and, therefore, do not exhibit changes in the expression of smile, emotional state and altruism behavior compared to individuals without depression.

## Results

### Cohort characterization

The participants were divided into the non-depressive (n = 69; 55 women; mean age = 24.29; SD = 3.4) and depressive (n = 16; 11 women; mean age = 25.6; SD = 4.08) groups. The groups were similar in age (t = 0.69; p = 0.48) and sex (p = 0.11). The depressive group showed higher levels of depression compared to the non-depressive group (p < 0.001). The values for the self-depreciation, affection and cognition, and somatic dimensions of the depression scale in the depressive group were significantly higher than those in the non-depressive group (Table 1).

**Table 1:** Sample characterization.

	Non-depressive, n = 69	Depressive, n = 16	p value*
Gender (F/M)	55/14	11/5	p = 0.11
Age (years old)	24.29 (3.4)	25.6 (4.08)	p = 0.48
Depression	10.82 (5.53)	26.7 (10.7)	p < 0.001*
F1. Self-denigration	3.5 (2.25)	8.23 (4.64)	p < 0.001*
F2. Affection and cognition	4.34 (2.84)	10.06 (4.33)	p < 0.001*
F3. Somatic	2.03 (1.44)	4.70 (1.68)	p < 0.001*

Data expressed as mean (SD).

*Statistically significant difference (p < 0.05).

### Social interaction pictures increase zygomatic major muscle activation in non-depressive but not in depressive individuals

The ANOVA showed a main effect for pictures block (F(2, 345) = 20.83, p < 0.0001), and time (F(11, 1683) = 11.74, p < 0.0001), but not for group (F(1, 125) = 2.69, p = 0.10). It also showed an interaction between group and block (F(2, 337) = 6.74, p = 0.001), group and time (F(11, 1,694,345) = 1.81, p = 0.047), and block and time (F(22, 1618) = 2.98, p < 0.0001), but it did not show a triple interaction among group, block and time (F(22, 1614) = 1.10, p = 0.34).

**Figure 1:**
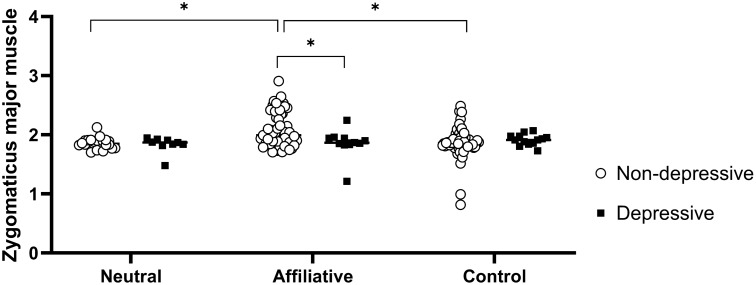
Least square means of electromyographic activation of the zygomatic major muscle in the non-depressive (white circles) and depressive (black squares) groups during the visualization of neutral, affiliative and control blocks. The points represent the mean value of each volunteer in microvolts transformed by Box-cox. *p < 0.05.

The Tukey–Kramer post hoc tests of the interaction between group and block showed that the non-depressive group had greater activation of the zygomatic major muscle during visualization of the affiliative block than the neutral (p < 0.0001) and control (p < 0.0001) blocks. In addition, zygomatic major muscle activation was greater in the non-depressive group than in the depressive group during visualization of the affiliative block (p = 0.02) (Fig. 1).

**Figure 2:**
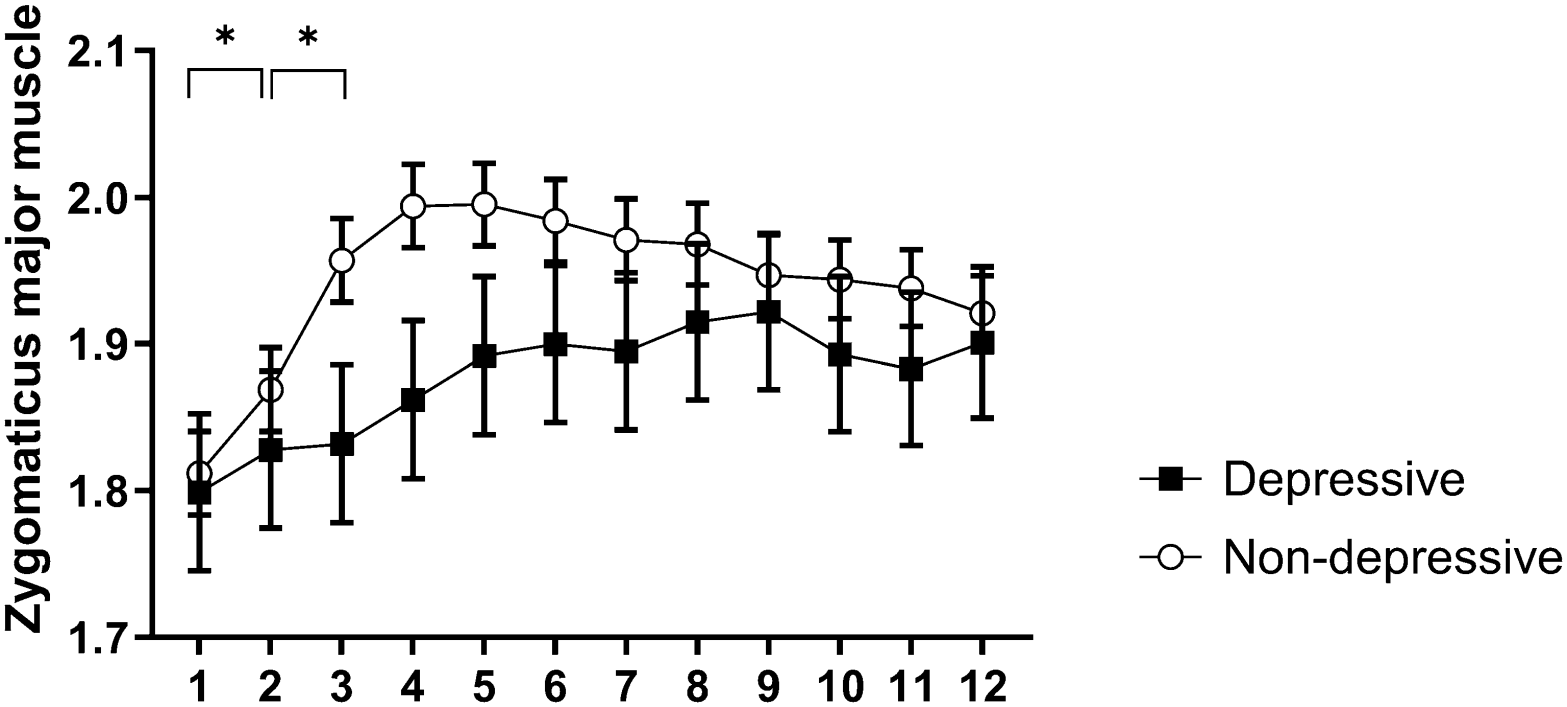
Temporal course of the least square means of electromyographic activity of the zygomaticus major over 12 half-seconds times in microvolts transformed by Box-cox considering the neutral, affiliative and control blocks collapsed for depressive (black squares) and non-depressive (white circles). *p < 0.05 represents the comparison between time 1 and 2, and time 2 and 3 in the non-depressive group.

With regard to the interaction between group and time, the post hoc tests showed greater activation of the zygomatic major muscle in the non-depressive group during the second time compared to the first (p = 0.02) and during the third time compared to the second (p < 0.001) (Fig. 2).

**Figure 3:**
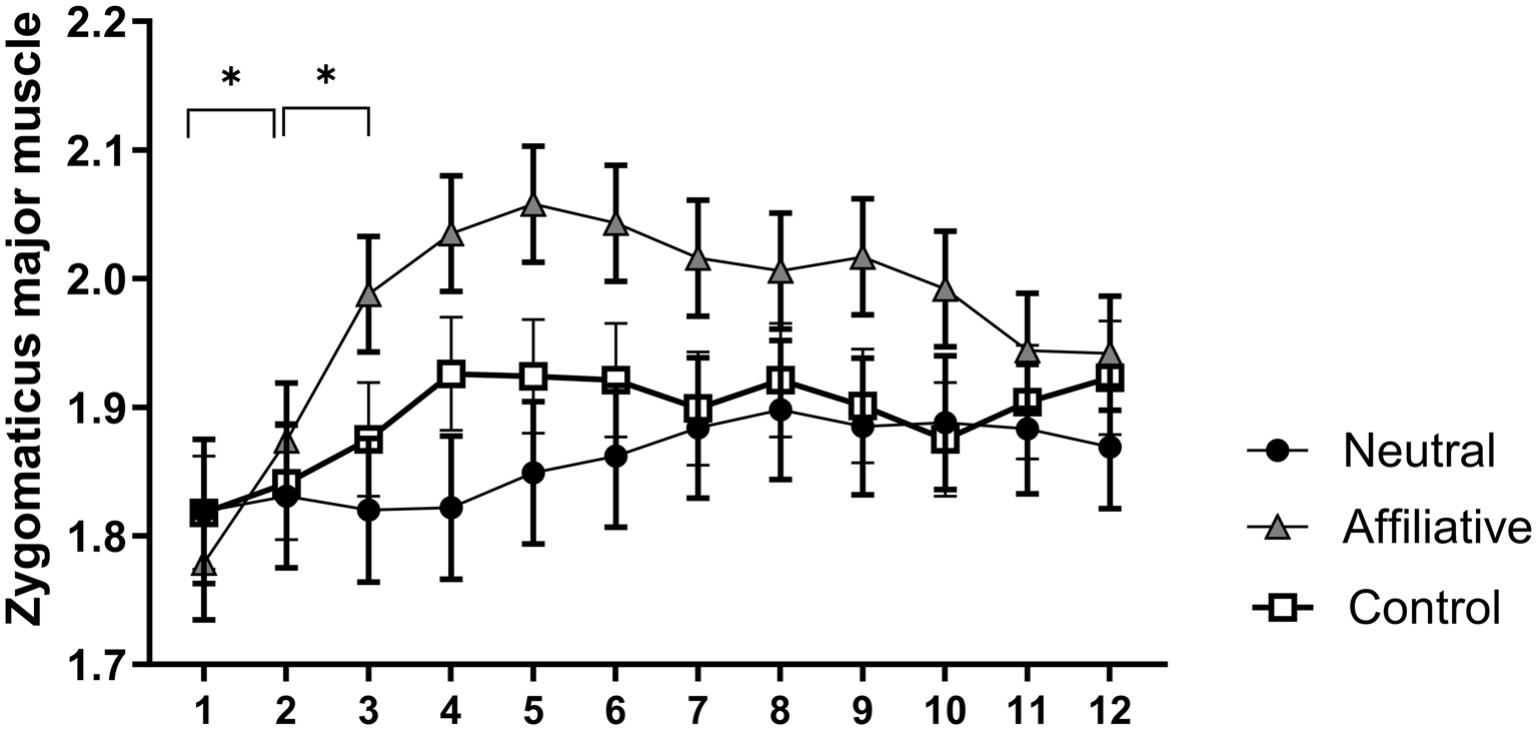
Temporal course of the least square means of electromyographic activity of the zygomaticus major over 12 half-seconds times in microvolts transformed by Box-cox. of neutral (black circles), affiliative (gray triangles) and control (white squares) pictures considering that both groups collapsed. *p < 0.05 represents the comparison between times 1 and 2, and times 2 and 3 for affiliative pictures.

Finally, in the interaction between block and time, the Tukey–Kramer tests showed that the affiliative block of pictures was associated with a greater activation of the zygomatic major muscle during the second time compared to the first (p = 0.049) and similarly when comparing the third time to the second (p = 0.002) (Fig. 3).

### Social interaction pictures reduce the fear of rejection in non-depressive individuals and increase it in depressive individuals

**Figure 4:**
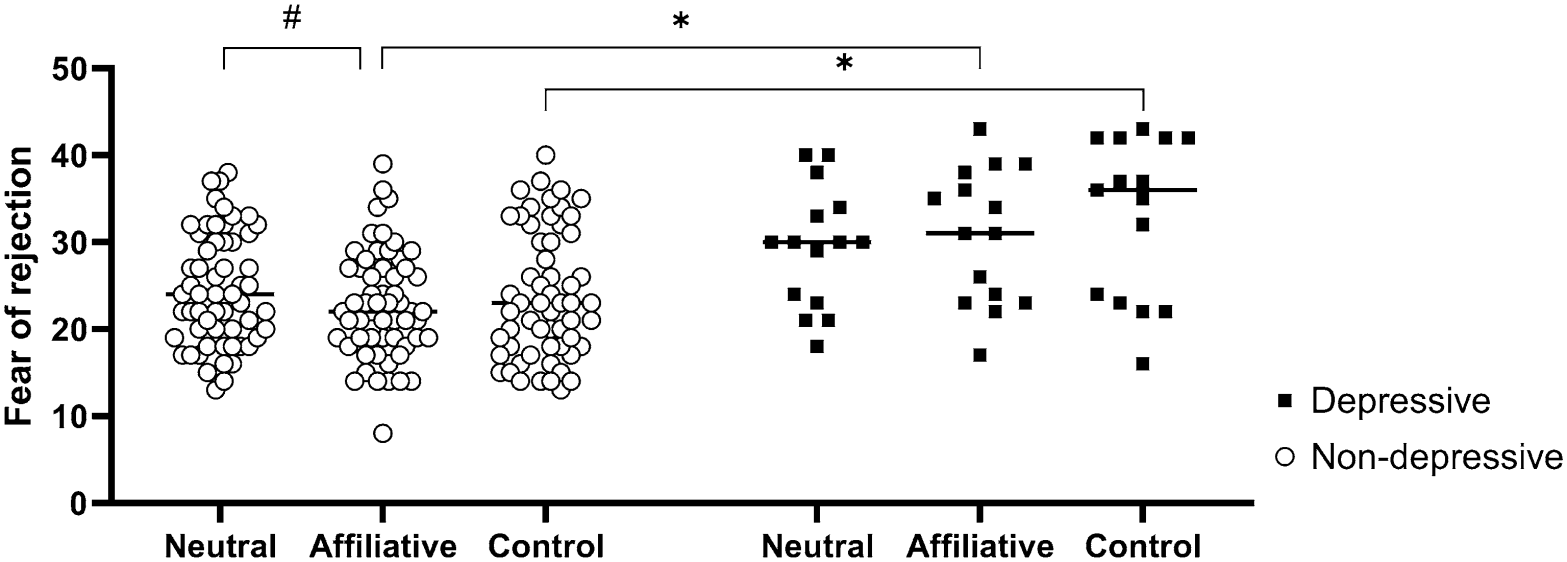
Fear of rejection scores after viewing the picture blocks. Self-reported values in the non-depressive (white circles) and depressive groups (black squares). The points represent the mean value of the fear of rejection score for each participant. *p < 0.05 and ^#^p = 0.06.

The ANOVA with the scale of fear of rejection showed a main effect of group (F(1, 71) = 15.41, p = 0.0002) and block (F(2, 142) = 3.53, p = 0.0 3) and an interaction between group and block (F(2, 142) = 4.92, p = 0.009). The Tukey–Kramer post hoc tests showed that fear of rejection marginally reduced after the affiliative block compared to the neutral block in the non-depressive group (p = 0.06). The fear of rejection was significantly greater in the depressive group than in the non-depressive group after the affiliative (p = 0.002) and control (p = 0.0004) blocks (Fig. 4).

The ANOVA with the scale of expectation of approximation showed a main effect of group (F(1, 71) = 9.87, p = 0.03), but no main effect of block (F(2, 142) = 2.68, p = 0.07) or interaction between group and block (F(2, 142) = 2.01, p = 0.14). The non-depressive group had more expectation of approximation than the depressive group (p = 0.03) independently of the block of pictures.

The ANOVA with the scale of altruistic behavior to friends showed a main effect of group (F(1, 69) = 4.00, p = 0.049), but no main effect of block (F(2, 138) = 0.40, p = 0.67) or interaction between group and block (F(2, 138) = 0.06, p = 0.94). The non-depressive group had more altruistic behavior to friends than the depressive group (p = 0.49) independently of the block of pictures.

The ANOVA with the scale of altruistic behavior to strangers showed no main effect of group (F(1, 69) = 1.90, p = 0.17) or block (F(2, 138) = 1.95, p = 0.15) nor for the interaction between group and block (F(2, 138) = 0.15, p = 0.86).

## Discussion

The present study evaluated the differences in EMG reactivity of the zygomatic major muscle and emotional states in depressive and non-depressive individuals upon exposure to visual stimuli that were neutral, with social interaction (affiliative), and without social interaction (control). An important control performed in the present study was that the blocks of pictures with and without social interaction were pairs of pictures showing the same people and background scene, with the same mean valence and activation. The only difference between the blocks was the context of social interaction. When viewing the affiliative pictures, the non-depressive individuals showed greater zygomatic EMG reactivity compared to the control and neutral pictures, thus demonstrating increased smile expression. In contrast, the depressive group showed no difference in EMG reactivity while viewing the three image blocks. In addition, greater sociability (less fear of rejection) was observed in the non-depressive group compared to those in the depressive group. These results suggested that individuals with depression have decreased smile and sociability to social interaction scenes compared to that in individuals without depression, which demonstrated their difficulty in reacting to pleasant stimuli with social content.

Higher valence stimuli increase the EMG activity of the zygomatic major muscle, while lower valence stimuli show lower activation of this musculature^7,19^. The results observed in non-depressive individuals corroborate those reported previously and are consistent with those of a study showing increased zygomatic activity for social interaction pictures (preceded or not by a priming text) compared to control pictures also pared by valence and arousal^9^. Additionally, the temporal analysis during image visualization showed that the affiliative pictures promoted increased smile expression from 0.5 s onwards compared to the control pictures, a difference that persisted until the end of the image visualization. Thus, the social interaction clues not only promoted greater smile expression but also induced a sustained positive emotional mood^9,20,21^.

Among the high-valence visual stimuli, those that evoked sociability, such as pictures of people interacting socially and pictures of babies, increased zygomatic EMG activity by activating brain circuits that “prepare” individuals for social interaction^22–25^. However, most studies on psychophysiological reactions using social interaction scenes are carried out in healthy individuals and do not present stimuli matched for valence and activation^4,26^. Therefore, when observing the increase in zygomatic EMG activity during exposure to pictures with scenes of social interaction (affiliative block) compared to pictures with the same pairs of people, but without social interaction (control block), the results of the present study suggested that the context of social interaction promoted a smile expression, which acted as a facilitator for social interactions in healthy people^9,27^. However, this effect did not occur in people with depression.

Depressed individuals show less smile expression than control individuals^28,29^. A reduction in zygomatic EMG activity was also observed during the visualization of high-valence pictures depicting happy faces, indicating that social pictures of high pleasantness provoke negative responses in individuals with depression compared to those in control individuals^4,30^. A review of 39 studies showed alterations in emotional facial expressions across different mental disorders. The majority of studies point towards decreased facial emotional expressivity in individuals with depression, specifically, the decrease in facial expression is mainly evident for positive stimuli^31^. Our results corroborate these findings and additionally show that individuals with depression are not responsive to highly pleasant social stimulus. In the present study, the affiliative and control image sets had similar valence and arousal, number of people and background scene; the only difference between the blocks was the social content. Thus, affiliative pictures promote changes in relevant facial expressions that drive social interactions^9^ but not in individuals with depressive symptoms.

Facial expressions have an important influence in social contexts, especially the smile, which can evoke feelings of affiliation. Zygomatic activity has been proposed to act as a social facilitator^32^ indicating a willingness to make connections^33–35^. We suggest that individuals with depression have low responsiveness to the social context, as reflected by apathy in their facial expressions. Therefore, this factor may be a determinant for poorer social interactions and avoidance of contact with other people among individuals with depression^13^.

Studies on the social interactions have reported sadness or anguish in the facial expressions of depressed individuals, including low zygomatic activity to avoid arouse sympathy, help, and proximity to those around them^36^. However, other studies emphasize that the interpersonal behaviors of individuals with depression lead to their rejection by the people with whom they live. The behaviors of people with depression are categorized as aversive, which, in turn, reinforces the cycle of loneliness and further accentuates the depression. While some studies claim that people with depression have low accuracy in correctly identifying happy faces, others^1,4,28,36^ observed that depressed individuals could differentiate pictures with happy expressions from pictures from other categories (for example, pictures with sad expressions), with similar precision to non-depressed individuals. However, the picture blocks in the present study were matched for valence and activation and had similar proportions of smiles (affiliative block 87.5%, control block: 71.4%); the only differences between them were the subtle cues of social interaction. Thus, we supposed that for individuals with depression, social interaction is not a pleasant enough stimulus, resulting in the lack of changes in smile expression.

Another component that could influence the facial expressiveness in depressed individuals is facial mimicry. Mimicry facilitates the creation and maintenance of sociability and plays an important role in social interactions seeing that it creates empathy, linking and affiliation between people^37,38^. Hess and Fisher suggested that mimicry functions as a social regulator^39,40^; thus, when mimicry is hindered or disturbed, it can impair emotion recognition^41^ and lead to elevated stress reactions in the interaction partner^42^. In a review, Kampf^43^ showed that mimicry is decreased in depressive states^44–46^, whereas mimicry is increased in positive mood states^46^. Research has shown that patients with depression show less mimicry of pictures of happy and sad faces compared to the non-clinical control group^47^, and symptom severity of depression is associated with fewer affiliative and higher non-affiliative facial expressions^29^. Moreover, acutely depressed patients compared to remitted patients and non-clinical participants showed less mimicry of happy faces and were less accurate in recognizing happy faces, yet reduced mimicry did not mediate these deficits^14^. This suggests that people with affective disorders might show less mimicry behavior during depressive episodes, which may in turn influence their social relationships^39^. After exposure to the affiliative block of pictures, the non-depressive group showed less fear of rejection compared to neutral block. No modulation was observed in the depressive group. Therefore, in the non-depressive group, the affiliative pictures motivated social interactions, increasing states of sociability (reducing the fear of rejection scores), an effect not observed in the depressive group. These results corroborate those described in the literature, which showed a reduced fear of rejection after viewing affiliative pictures in non-depressive individuals^9,23^. It is known that people with depression often have the expectation that they might be rejected or that it is too exhausting to engage with others^48,49^, which may also lead to less mimicry behavior and less states of sociability. However, to the best of our knowledge, this result has not been demonstrated in people with high levels of depressive symptoms during the visualization of positive social stimuli.

Our results can also be explained by the “social risk hypothesis” of depression^29,50^. This hypothesis proposes that people with high levels of depressive symptoms tend to distance themselves from other people to protect themselves from rejection, contempt, and social exclusion. This happens through the signaling of submission in socially competitive environments, in addition to distancing in exchange-oriented social contexts, where requests for help could be ignored or ridiculed^28^. As depressive symptoms disappear, individuals send more signals that indicate their willingness to interact socially^50^. Our results are consistent with this hypothesis, in which the affiliative pictures did not reduce the fear of social exclusion, in contrast to the observations in the non-depressive group. Furthermore, according to the social risk hypothesis, help-seeking behavior is reserved for contexts oriented towards reciprocity with friends and family, who are more likely to provide the requested help^29,50^. Since our stimuli comprised of pairs of pictures depicting individuals unknown to the volunteers, we can assume that this may also have contributed to the non-modulation of affiliative behaviors in individuals with depression, as they tend to direct their affiliative behaviors to known people in whom the risk of exclusion is minimized^29,50^. Adaptations in future experiments should be considered to assess the responsiveness of individuals with depression exposed to visual stimuli from people in their social circle, as described by Ref.^51^ in a non-depressed sample.

Altruistic behaviors are defined as voluntary actions performed without interest in receiving internal or external rewards and intended to improve the well-being of others^52,53^. We expected that non-depressive group would increase the altruistic behavior after visualizing the affiliative block. But in the non-depressive and depressive group, no changes were observed in altruistic behavior towards friends or strangers.

The present study has some limitations. First, our sample, despite high scores on the BDI-II, is not a clinical sample and the participants were not diagnosed with depression based on the diagnostic and statistical manual of mental disorders, Fifth Edition (DSM-5) by a psychiatry^54^. Therefore, future studies in a clinical sample should be considered. Secondly, although the groups had the same internal proportions of men and women, the sample mostly comprised women. Additional studies with balanced sex distributions in the sample overall are needed. Thirdly, our groups are unbalanced. We have much more non-depressive participants. Fourthly, our visual stimuli had a low ethnic diversity. As the feeling of belonging to a group favors facial mimicry^55^, visual stimuli that cover all ethnic diversity are needed to increase participant sense of belonging in the experimental context.

We concluded that individuals with high levels of depressive symptoms are not susceptible to emotional modulation promoted by social interaction cues; that is, they are not able to promote the sustained expression of a smile and to increase their feelings of sociability. Through self-report and facial EMG measurements, we showed that affiliative pictures increased the somatic responses of smile expression and reduced the fear of being rejected in individuals without depressions, in contrast to individuals with high levels of depressive symptoms, who did not present these responses.

## Methods

### Participants

The cohort comprised 85 individuals (66 women; 77%) aged between 18 and 35 years (average = 23.9, SD = 3.7). The exclusion criteria were: a medical diagnosis of psychiatric diseases; history of peripheral or central facial paralysis; and use of continuous medications (except for contraceptives).

The participants answered the Beck Depression Inventory II (BDI-II)^56^, and based on the final score, the participants were separated into groups without symptoms of depression (non-depressive group: n = 69) and with symptoms of depression (depressive group: n = 16). The study followed the recommendations of the Declaration of Helsinki and the experimental protocol was approved by the Research Ethics Committee of the Federal University of Ouro Preto – Brazil (CAAE 90012318.10000.5150). All participants provided written informed consent. Data were collected before the COVID-19 outbreak.

### Visual stimuli

The participants were exposed to three blocks containing 28 pictures each (neutral, affiliative and control). The neutral block consisted of neutral pictures taken from the *International Affective Picture System* (IAPS)^25^ (valence: mean = 4.96; SD = 0.49; activation: mean = 2.76; SD = 0.8). The pictures of the affiliative and control blocks were extracted from an image^8^ and their valence and activation values were classified as described by Ref.^25^. These blocks depicted pairs composed of an adult and a child, or two children. For each image in the affiliative block depicting two individuals interacting socially, there was an image in the control block that portrayed the same pair of individuals, in the same scenario, nevertheless without direct social interaction between them. The affiliative and control blocks had approximately the same number of smiles (affiliative: 87.5% and control: 71.4%) and were paired by valence (affiliative: average = 7.17; standard deviation = 0.39; control: average = 7.02; standard deviation = 0.38; t = 1.39; p = 0.17) and activation (affiliative: average = 3.69; standard deviation = 0.50; control: average = 3.92; standard deviation = 0.59; t = − 1.58; p = 0.41). The block order was fixed across participants to reduce the number of sequences and because we previously tested the block difference in a similar experimental design^9^. The pictures taken from the IAPS catalog were: 2880; 5510; 5520; 6150; 7000; 7002; 7004; 7006; 7009; 7010; 7025; 7050; 7080; 7130; 7090; 7150; 7170; 7175; 7207; 7211; 7217; 7233; 7235; 7490; 7550; 7595; 7705; and 7950. For examples of pictures affiliative and control block, see Refs.^8^ and ^9^.

### Psychometric assessments

The participants completed the following questionnaires:

BDI-II^56,57^: This 21-item self-report measure assesses depressive symptoms in the cognitive, affective, somatic, and motivational dimensions. The BDI-II scores range from 0 to 64, with the choice of cut-off point depending on the sample and study objectives^58,59^. In clinically undiagnosed samples the term “depression” should be used only for scores above 20, preferably with a concomitant clinical diagnosis^59–61^. Thus, in the present study, individuals with scores above 21 were included in the depressive group^59,60^.

*Affiliative status scale*^62^. This scale comprises 27 adjectives that are divided into two subscales: 13 adjectives form the expectation of approximation subscale, which assesses the individual’s motivational state in performing social interactions, and 14 adjectives form the fear of rejection subscale, which predicts the motivation to be kept away from contact with others. The scores on the two subscales range from 13 to 52 points for the expectation of closeness and 13 to 52 points for the fear of rejection.

*Altruistic behavior scale*^63^. This scale includes 16 items, 8 of which assess the individual’s altruistic behavior towards a friend and eight which assess behavior towards a stranger. The score for each subscale ranges from 0 to 32 points. The final score is the sum of the two subscales, defined as the total altruism score, where the higher the index, the higher the individual’s level of altruism.

### Display of visual stimuli and signal processing

Visual stimuli were displayed on a 23″ S23C550H Samsung TV monitor placed 94 cm in front of the participants. All pictures were viewed full-screen. E-Prime version 2.0 (Professional Psychology Software Tools Inc., Pittsburgh, PA), was used to generate the visual stimuli shown on the screen and to generate the pulses related to the beginning of these stimuli. The triggers generated by E-Prime were sent by parallel cable to the EMG signal acquisition system. A BIOPAC MP100 amplifier (Biopac Systems, Inc., Goleta, CA; https://www.biopac.com/) was used, with a sampling rate of 1000 Hz and a gain of 1000 for the EMG100C electromyographic module.

The EMG signal was filtered online using a 10-Hz high-pass filter and a 500-Hz low-pass filter. The amplifier was connected directly to Acknowledge software (https://www.biopac.com/) to acquire the raw electromyographic signals in real time. The raw signals were pre-processed off-line in MATLAB version 7.0 (MATricesLABoratory). We applied a 2th-order Butterworth filter with a cutoff frequency of 20 Hz followed by a constant detrending to remove artifacts in the EMG signal. After we used the full-wave rectified signal. Spreadsheets with the signs of each volunteer were prepared in Microsoft Excel 2016.

The total window analysis of zygomaticus major muscle was defined from − 2 to 6 s. Mean amplitude of activation of EMG muscle activity in the interval between − 2 s and time zero (beginning of the stimulus) was used as the baseline and was applied for baseline correction. Mean amplitude of activation of EMG muscle activity in the interval between 0 and 6 s (picture visualization) in bins of 500 ms was used as the picture windows analysis, totaling 12 intervals. Processing was performed as described by Refs.^9,20,21^.

### Experimental procedure

All procedures were performed in a room with controlled lighting, acoustic isolation, and controlled temperature (21° C). The participants remained seated in a comfortable armchair positioned in front of a table upon which was placed a computer monitor. To preserve privacy and avoid discomfort when completing the scales, the participants remained alone in the room while completing the questionnaires and viewing the pictures.

The participants started the experiment by completing the BDI-II^56^. Afterward, each participant was asked to clean their face with running water and neutral soap and were advised to use the bathroom and drink water, if desired. Then, the area on which the electrodes were placed on the face of the participants was cleaned with 70% alcohol and a small exfoliation with a paper towel was performed. To collect the EMG signal, two 4-mm silver chloride (Ag–AgCl) electrodes were placed on the zygomatic major muscle on the left side of the face, as described by Ref.^64^.

**Figure 5:**
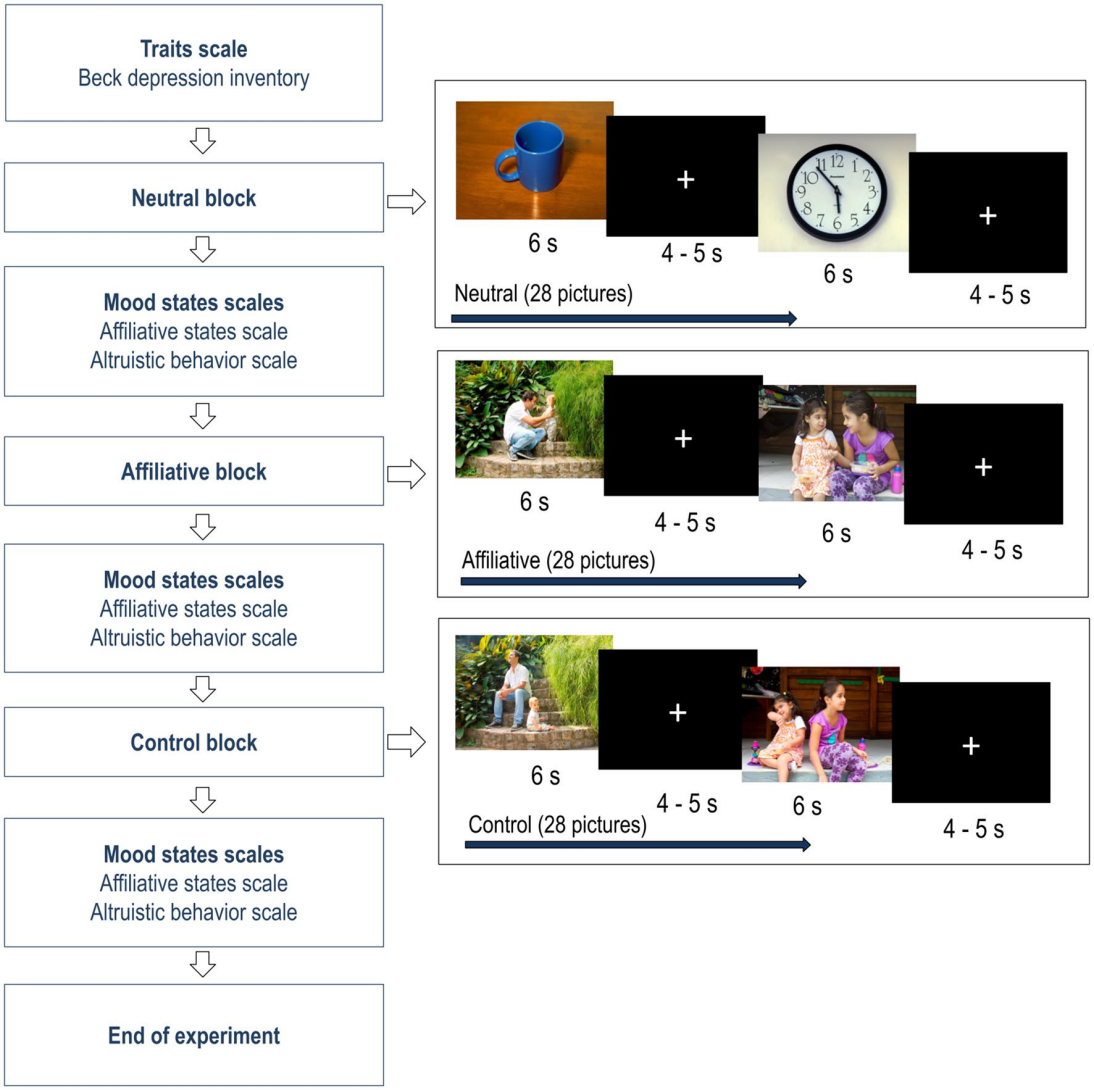
Sequence describing the order of the events throughout the experiment. The two experimental groups were subjected to the same sequence of experiments. Initially, they completed the scale of depression. In sequence, the participants started viewing the 28 pictures of the neutral block, 28 pictures of the affiliative block, and 28 pictures of the control block. At the end of each block of pictures, the volunteers completed the affiliative state scales and the altruistic behavior scale. The state scales were applied a total of three times (once after each block). All pictures were displayed for 6 s. Between each image, a black screen with a cross was displayed for 4–5 s. For more details about the complete picture catalogue and its standardization, see Silva et al.^8^. All pictures from the catalogue are property of the present research group, and their reproduction is authorized for scientific purposes only.

The participants received brief instructions on the dynamics of the experiment and were informed that all guidance regarding the procedures would appear on the computer screen. To ensure that the participants’ attention was maintained on the stimuli, the participants were instructed to keep their gaze fixed on the central point and carefully observe the pictures without looking away. Each block contained 28 pictures and each picture was displayed for 6 s, separated by a black screen, with a fixation point, which was displayed for a random duration (4–5 s). The experiment began with the presentation of the 28 pictures of the neutral block, followed by the blocks of interest; namely, the affiliate and control blocks. Between each block was an interval during which the participants responded to the mood state scales (affiliative state and altruistic behavior scales). After completing these scales, the display of pictures were continued, thus totaling 3 blocks and three applications of the mood scales. All three blocks had the same time settings. The duration of each of the three blocks varied between 280 s (4 min 40 s) and 308 s (5 min 8 s). At the end of the experimental session, the electrodes were removed and each participant was thanked. See Fig. 5 for the experimental sequence.

### Statistical analyses

Statistical analyses were performed using the web-based development environment of SAS software (SAS Institute Inc., 2015), version 9.04, and the graphs were plotted using GraphPad Prism 6.0.1 (GraphPad Software, Inc.).

For sample characterization, the two groups were compared using t-tests (age and symptoms of depression) and chi-squared test (gender).

The EMG data and the Mood State Scale data were analyzed using repeated measures models (ANOVA) and residual dependence was modeled considering a compound symmetry structure, which is typical of repeated measures data analysis. In the case of the Mood State Scale data, a model was used for each scale (expectation of approximation, fear of rejection, altruistic behavior to friends, altruistic behavior to strangers) considering group (depressive and non-depressive) as the between-subjects factor, and block (neutral, affiliative and control) as the within-subjects factor.

For the EMG data, a model was used considering group (depressive and non-depressive) as the between-subjects factor, and block (neutral, affiliative and control) and time (12 bins of 0.5 s) as the within-subjects factor. In this repeated measures model, interactions among these three factors were considered, and, since the number of times within a block was relatively high (12), it was found more appropriate to model residual dependence by means of an autoregressive structure of order 1. Under this structure, the residual covariance between the two time points tends to be lower as the time points are farther apart. This and other structures of residual dependence are easily handled in the 'Mixed' procedure of the SAS software. Moreover, for EMG data, the Box-Cox transformation^65^ was applied, using the boxcox function of the MASS package^66^ of the R software. For a given statistical model, the Box-Cox method estimates the best power transformation such that the distribution of residuals approaches a normal distribution.In both repeated measures models (for Mood State Scale and EMG data), whenever a pertinent null hypothesis was rejected (of an interaction, or of a single factor that does not interact with other factors), a post hoc multiple comparison test was carried out using the Tukey–Kramer method of adjustment. This method controls the familywise error rate, considering all pairs of means to be compared as a family of comparisons^67^.

The level of significance adopted in this study was α = 0.05.

## Data Availability

The datasets used and/or analyzed during the current study are available from the corresponding author on reasonable request.
